# Magnetic core–shell Carrageenan moss/Fe_3_O_4_: a polysaccharide-based metallic nanoparticles for synthesis of pyrimidinone derivatives via Biginelli reaction

**DOI:** 10.1186/s13065-018-0477-3

**Published:** 2018-10-27

**Authors:** Hossein Mohammad Zaheri, Shahrzad Javanshir, Behnaz Hemmati, Zahra Dolatkhah, Maryam Fardpour

**Affiliations:** Heterocyclic Chemistry Research Laboratory, Department of Chemistry, Iran University of Science and Technology, Tehran, 16846-13114 Iran

**Keywords:** Biopolymers, Biocatalyst, Carrageenan moss, Magnetic core–shell nanoparticles, Pyrimidinone, Biginelli reaction

## Abstract

Magnetically recoverable polysaccharide-based metallic nanoparticles Carrageenan moss/Fe_3_O_4_ (Fe_3_O_4_@CM) was tested for the synthesis of Pyrimidinone derivatives via Biginelli reaction under reflux conditions in Water. Interestingly, Fe_3_O_4_@CM prepared from unmodified Irish moss showed remarkable catalytic activity and recyclability. Low catalyst loading, simple reaction procedure, and using a green catalyst from a natural source are the important merits of this protocol.

**Electronic supplementary material:**

The online version of this article (10.1186/s13065-018-0477-3) contains supplementary material, which is available to authorized users.

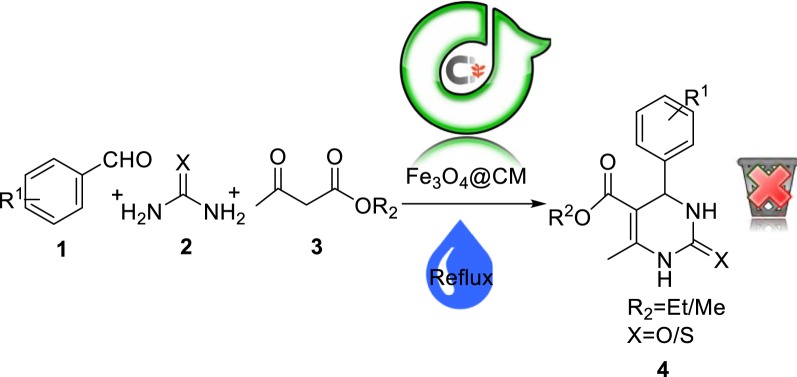

## Introduction

**Fig. 1:**
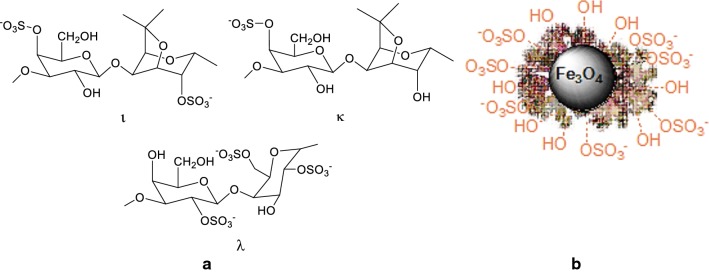
The structures of iota-, kappa- and lambda-carrageenan (**a**) and Fe_3_O_4_@CM (**b**)

The environmental factor is now the basis for new industrial processes. It covers not only the atom economy, but also the solvent economy and the energy consumption, as well as reducing the costs and chemical risks. One of the current defies of industrial research is to bring all these principles to discover effective and environmentally friendly synthetic methodologies. For all these reasons, today, most chemical methods of synthesizing pharmaceutical compounds, food or cosmetics are designed to make benefit of catalytic systems. One of the major challenges of a catalytic post-treatment process is the development of less expensive and more environmentally friendly catalysts. In this context, heterogeneous catalysts offer an answer to these problems by being easily separable from the reaction medium and in some cases reusable. In this regard, the use of magnetic nanoparticles has emerged as a feasible solution; their insoluble and paramagnetic nature enables easy and efficient separation of the catalysts from the reaction mixture with an external magnet. On the other hand, the magnetically retrievable nanocatalysts provide immense surface area, excellent activity, selectivity, recyclability and long lifetime [1–3]. Of the iron oxides only maghemite (γ-Fe_2_O_3_) and magnetite (Fe_3_O_4_) display ferrimagnetism due to the spinell structure. The naturally occurring magnetic compound clearly contains many interesting properties and potential for various applications and is commonly used in the composition of heterogeneous catalysts [4]. Various approaches exist for magnetic nanocatalysis, the mainstream of which uses the nanoparticle simply as a vehicle for recovery, to which a protective coating, then a metal binding ligand is anchored at the cost of much synthetic effort. By such a method, one could envisage anchoring nearly any homogeneous catalyst to a magnetic particle, so this method has a very broad scope of potential reactions. The utilization of polymer-coated magnetic particles and polysaccharide-based bio-nanocomposites is currently of particular interest; especially the ones composed of natural polymers that has become a very interesting approach in nanocatalytic protocols. Natural polysaccharides are important types of biopolymers with excellent properties due to their chemical and structural diversity [5]. The marine environment and the diversity of associated organisms, offer a rich source of valuable materials. Amongst the marine resources, polysaccharides of algal origin include alginates, agar and carrageenan are well known natural sources of polysaccharides. The three main varieties of carrageenans are iota (ι-), kappa (κ-) and lambda (λ-). Their structures are shown in Fig. 1a. The presence or absence of 3,6-anhydro-d-galactose bridge, the number and the position of the sulphate substituents on the galactose carbons make it possible to classify the different categories of these polymers. Agri-food industry is considered as the main user of carrageenans. For instance, Kappa- and iota-carrageenans are used as gelling agents, and lambda-carrageenans as thickeners. The industrial source of carrageenan is *Chondrus crispus* (Irish moss or Carrageen moss), a species of red algae that grows abundantly along the rocky parts of the Atlantic coast of Europe and North America. Irish moss (IM) is mostly composed of proteins (~ 50%), carbohydrates (~ 40%) and inorganic salts (~ 10%). The water-soluble extract of Irish moss, also known as carrageenan, is a hydrocolloid gum rich in sulfated polysaccharides, with 15–40% sulfate ester content and a relative average molecular weight well above 100 kDa [6, 7]. Therefore, we decided to evaluate the catalytic activity of natural marine-derived polymer carrageenan and magnetically Fe_3_O_4_ nanoparticles, Fe_3_O_4_@CM (Fig. 1b) as a novel nano-biocatalyst in synthesis of some valuable heterocyclic compounds.

**Fig. 2:**
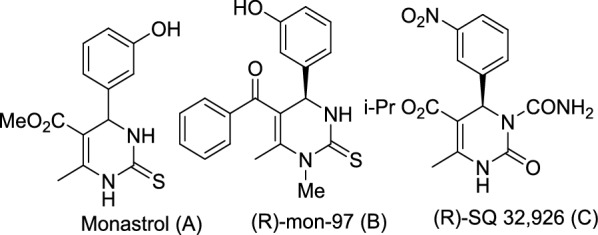
Representative natural products DHPMs-containing framework

In the last two decades, a large number of reports and reviews have dealt with the development and enhancement of the reaction conditions for the synthesis of 4-dihydro-2(H)-pyrimidinones (DHPMs) [8]. DHPMs are pharmacophoric templates that can exert potent and selective actions on a diverse set of membrane receptors, including ion channels, G protein-coupled receptors and enzymes, when appropriately substituted. They are thereby, valuable building blocks for the synthesis of important heterocyclic derivatives and possess a broad range of biological and pharmacological activities including the first cell-permeable antitumor scaffold, Monastrol (A), the modified analogue (R)-mon-97 (B) and anti-hypertensive agent (R)-SQ 32,926 (C) (Fig. 2) [9–11]. Given that the original reaction conditions suffered from certain drawbacks, such as low yields and limited scope, using various catalysts and numerous alternative substrates under different reaction conditions, has improved the synthesis of a vast number of DHPM derivatives with enhanced yields.

**Scheme 1:**
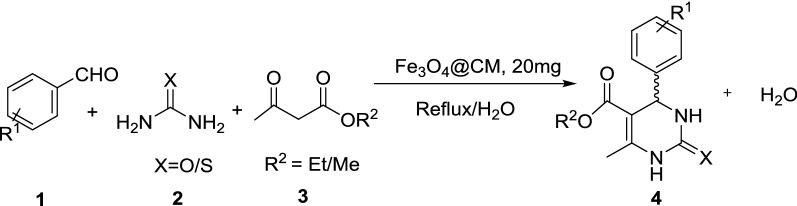
Synthesis of substituted pyrimidines catalyzed by Fe_3_O_4_@CM

In continuation of our previous work based on the preparation and application of magnetically recoverable nano-biocatalysts Fe_3_O_4_@CM in MCRs [12], we decided to evaluate the catalytic activity of natural marine-derived polymer carrageenan and magnetically Fe_3_O_4_ nanoparticles, Fe_3_O_4_@CM (Fig. 1b) as a novel nano-biocatalyst in the synthesis of functionalized 3,4-dihydro-2(H)-pyrimidinone (DHPM) derivatives via Biginelli reaction, a one-pot cyclocondensation of a β-keto ester, urea/thiourea and an aromatic aldehyde, using a Brønsted acid–base solid catalysis (Scheme 1).

## Results and discussion

### Characterization of Fe_3_O_4_@CM

**Fig. 3:**
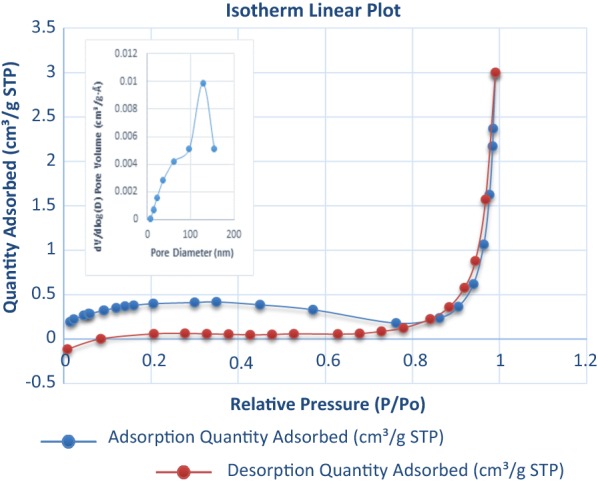
BET surface area analysis

The catalyst was synthesized and characterized according to our previous method [12]. The synthesized magnetite nanoparticles were characterized by various techniques, such as FT-IR spectroscopy, scanning electron microscope (SEM), energy-dispersive X-ray spectroscopy (EDX), Transition electron microscope (TEM), thermogravimetric analysis (TGA), vibrating sample magnetometer (VSM) analysis (see Additional file 1), and Brunauer–Emmett–Teller (BET) surface area analysis. The specific surface area, total pore volume (TOPV) and average pore diameter were obtained by N_2_ adsorption isotherms calculated by BET and BJH methods and found to be 1.2209 m^2^/g, 0.004168 cm^3^/g, and 54.1501 nm (Fig. 3). N_2_ sorption isotherms of the sample resembled Type IV isotherms, indicating the presence of mesopores (textural porosity) [13].

**Fig. 4:**
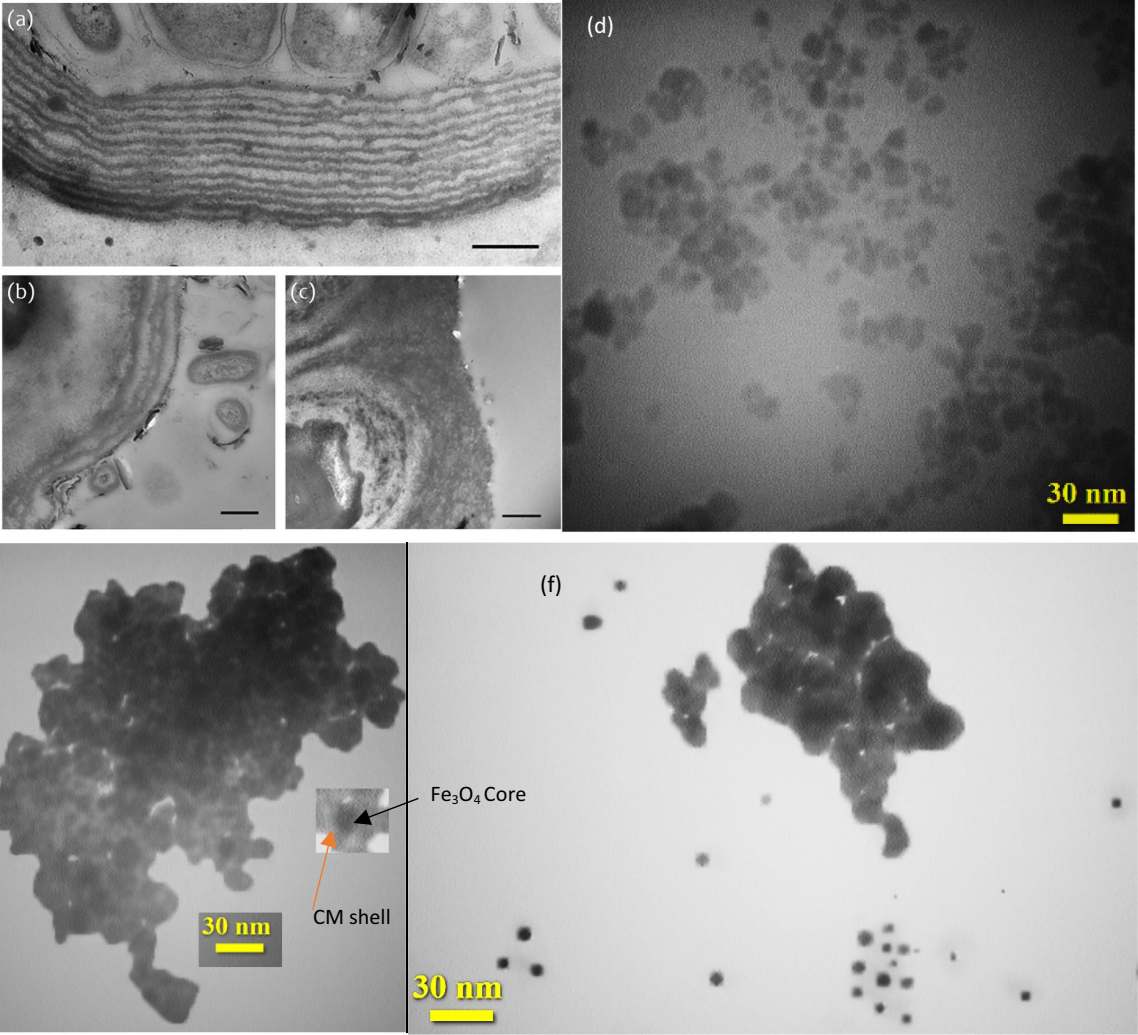
TEM micrographs showing the cuticle of a *Chondrus crispus* frond at sections from **a** tip, **b** middle and **c** base (Reprint by permission from www.nature.com/scientificreports 10.1038/srep11645) and **d**–**f** Fe_3_O_4_@CM with 30 nm magnification

The TEM micrographs (a, b, and c) of Carrageenan moss (*Chondrus crispus*) and Fe_3_O_4_@CM (d, e, f, and g) are shown in Fig. 4. TEM images reveal the spherical shape of nanoparticles with a diameter of about 15 nm, and clearly divulge the core–shell structure of Fe_3_O_4_@CM, with an average core diameter of about 10 nm, and CM shell thicknesses ranging from 3 to 5 nm.

### Optimization of the reaction conditions

To evaluate the catalytic activity of Fe_3_O_4_@CM for the synthesis of pyrimidinone derivatives, a combination of 4-chlorobenzaldehyde (**1a**), urea (**2a**) and ethyl acetoacetate (**3a**) (1:1:1 mol ratio) was considered as the model reaction. The obtained results are presented in Table 1. Under catalyst-free and reflux conditions in water, a trace amount of the desired product **4a** was formed after 3 h (Table 1, entry 1). An excellent 87% yield of **4a** was formed after 1.5 h when the reaction was carried out in the presence of 10 mg of the catalyst (Table 1, entry 2). To explore the effect of reaction temperature, the reaction was performed at room temperature in water. The yield of the product decreased with the diminution of temperature (Table 1, entry 3). Next, in order to explore the effect of solvent on the product formation, the reaction was carried out under solvent-free conditions as well as using various solvents, such as EtOH, DMF, EtOAc, CHCl_3_ and Toluene (Table 1, entry 6–10). The best results were obtained in water under reflux conditions (Table 1, entry 2). Due to the superior effect of ultrasonic homogenization to mechanical agitation [13], the use of ultrasound was also investigated in water using an ultrasonic probe. When ultrasonic irradiation was applied to the reaction mixture at room temperature (Table 1, entry 5), the yield was comparable to that obtained under reflux conditions in water (Table 1, entry 2). Increasing the catalyst loading from 10 to 20 mg, led to an enhancement of the reaction yield and a decrease in the reaction time (Table 1, entry 11). Increasing the catalyst loading up to 30 mg did not affect the yield of the reaction (Table 1, entry 13). When the reaction was carried out under ultrasonic irradiation using 20 mg of the catalyst (Table 1, entry 12), the obtained yield did not compete with the one under reflux conditions. The non-magnetic Carrageenan moss (NMCM) also showed good catalytic activity (entry 14) but the reaction time was longer (almost twice) and the catalyst separation was not as easy as Fe_3_O_4_@CM. This observation can be explained by the size of the nanoparticles, their good dispersion and improved surface area.

**Table 1:** Optimization of the reaction conditions (catalyst loading, solvent and temperature) for the synthesis of **4a**

Entry	Condition/solvent	Catalyst (mg)	Temp (°C)	Time (min)	Yield (%)
1	H_2_O	0	100	180	Trace
2	H_2_O	10	100	90	87
3	H_2_O	10	25	360	64
4	SF	10	50	240	70
5	Ultrasound/H_2_O	10	25^b^	90	85
6	EtOH	10	78	120	73
7	DMF	10	153	180	67
8	EtOAC	10	77	150	80
9	CHCl_2_	10	61	240	63
10	Toluene	10	111	270	65
11*	*H*_*2*_ *O*	*20*	*100*	*60*	*95*
12	Ultrasound/H_2_O	20	25	60	75
13	H_2_O	30	100	60	95
14^a^	H_2_O	NMCM (10)	100	110	90

*Optimum reaction conditions

^a^The reaction was catalyzed by 10 mg of non-magnetic Carrageenan moss

^b^The temperature was kept at 25 °C using a water bath

### The scope of the substrates

To inspect the extent of the catalyst application, the condensation reaction of a variety of aldehydes with 1,3-dicarbonyl compounds (ethyl acetoacetate, methyl acetoacetate and acetylacetone) and urea or thiourea was also investigated under the optimal reaction conditions and the results are given in Table 2. In all cases, Fe_3_O_4_@CM smoothly catalyzed the reaction in green reaction media to form the corresponding DHPMs with high to excellent yields of 73–95%. Aromatic aldehydes with electron-donating groups such as 4-methyl-benzaldehyde, 4-chloro-benzaldehyde, and 4-methoxy-benzaldehyde were converted to the corresponding DHPM derivatives in high yields in reaction with 1,3-dicarbonyl compounds (ethyl acetoacetate, methyl acetoacetate and acetylacetone) and urea (Table 2, entries 1, 2, 3, 7, 8, 9, 11 and 12). Aromatic aldehydes bearing electron-withdrawing groups including 3-nitro-benzaldehyde and 2-nitro-benzaldehyde also gave the desired products in excellent yields under the same reaction conditions (Table 2, entries 4, 5 and 13).

**Table 2:** Synthesis of pyrimidine derivatives under optimum reaction conditions*

Entry	R^1^	X	R^2^	Product	Time (min)	Yield (%)	Mp (°C)	
							Observed	Reported [Refs]
1	4-Cl	O	Et		60	95	210–212	213–214 [14]
2	4-Me	O	Et		90	73	213–215	214–217 [15]
3	4-OMe	O	Et		90	87	200–202	202–203 [16]
4	2-NO_2_	O	Et		60	85	220–221	220 [17]
5	3-NO_2_	O	Et		45	76	214–216	217 [18]
6	H	O	Me		60	87	210–212	207–210 [19]
7	4-Cl	O	Me		60	85	205–207	204–206 [20]
8	4-OMe	O	Me		45	93	190–192	191–193 [19]
9	4-Cl	S	Me		60	90	154–155	153–156[21]
10	H	S	Me		60	90	225–227	226–228 [22]
11	4-Cl	S	Et		45	93	190–192	188–190 [21]
12	4-OMe	S	Et		60	88	152–154	151–153 [22]
13	3-NO_2_	S	Et		60	90	205–207	202–204 [23]

*Reaction catalyzed by Fe_3_O_4_@CM (20 mg) under reflux conditions in water

**Fig. 5:**
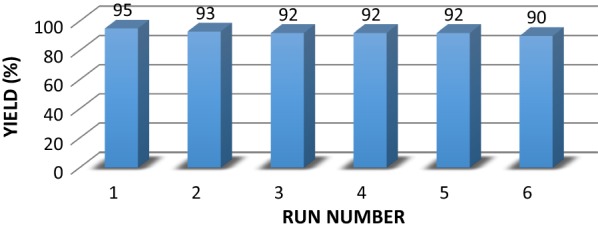
Reusability of Fe_3_O_4_@CM in the synthesis of pyrimidinones (**4a**)

In the next step, the recyclability and reusability of the catalyst were investigated. Upon completion of each run, the catalyst was collected with an external magnet, washed several times with ethyl acetate and ethanol, dried and used in the next run. The product yields were maintained high up to the sixth run (Fig. 5).

**Fig. 6:**
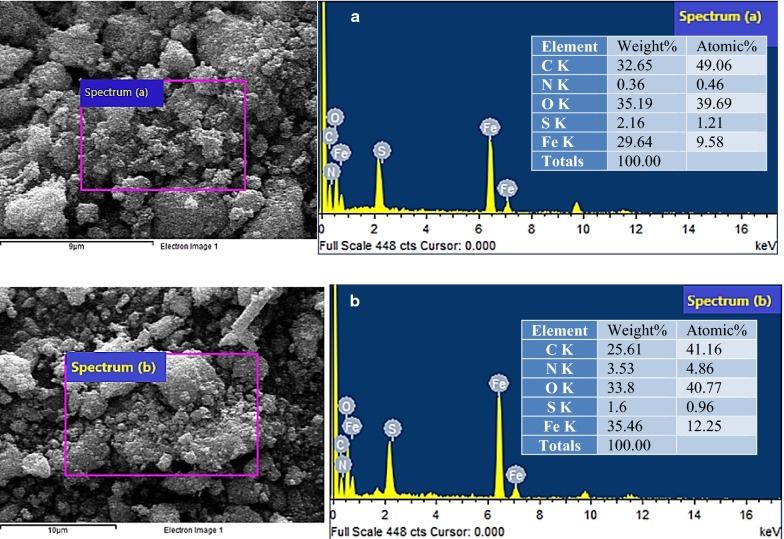
SEM and EDX analysis of Fe_3_O_4_@CM **a** before reaction **b** after recycling

Figure 6 shows the SEM micrograph, along with the corresponding elemental mapping and spectra by EDX, of a selected region of the fresh (Fig. 6a) and recycled Fe_3_O_4_@CM catalyst (Fig. 6b). As revealed by the EDX patterns, the Fe:S atom ratio has augmented from 8:1 in the fresh catalyst to 12:1 in the recycled catalyst. Therefore, there has been a 0.25% decrease in the atomic percentage of sulfur after recycling (Fig. 6b), which could explain the yield decrease during the consecutive catalytic cycles.

### Proposed reaction mechanism

**Scheme 2:**
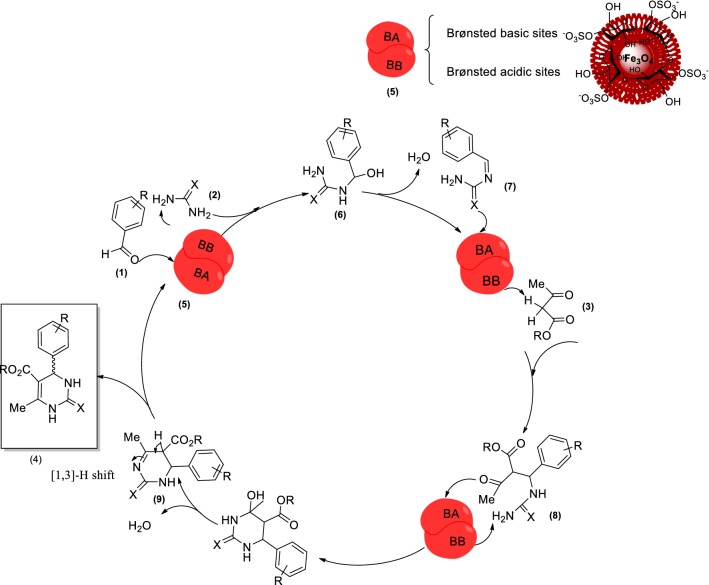
A plausible reaction mechanism for Fe_3_O_4_@CM-catalyzed Biginelli condensation reaction

A plausible reaction mechanism for the synthesis of DHPMs catalyzed by Fe_3_O_4_@CM is proposed in Scheme 2. *N*-acyl/thionyl iminium intermediate (**7**) is generated via cyclocondensation of aldehyde (**1**) and urea/thiourea (**2**) in the presence of Fe_3_O_4_@CM as a bifunctional Brönsted acid–base solid catalyst. Subsequently, 1,3-dicarbonyl compound (**3**) enters the reaction cycle, followed by cyclization and dehydration procedures under the acidic conditions to produce intermediate (**9**). Finally, a [1, 3] -H shift leads to the formation of the corresponding 3,4-dihydropyrimidin-2(1*H*)-one/thione (**4**).

**Fig. 7:**
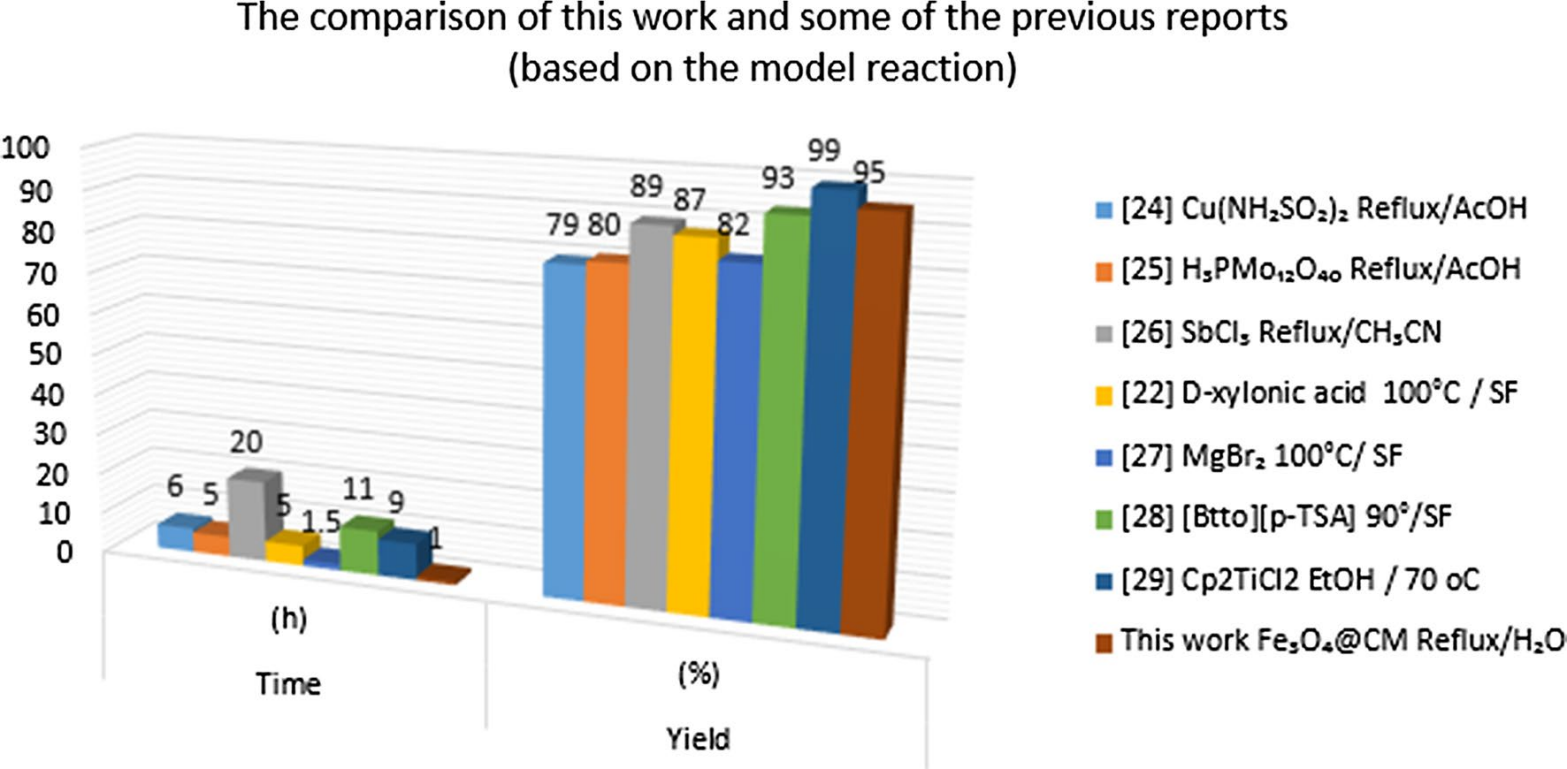
The comparison of this work and some of the previous reports using various catalysts under different reaction conditions

To demonstrate the effectiveness of Fe_3_O_4_@CM, a comparison of the present study and previous reports is illustrated in Fig. 7 [22, 24–29]. The results clearly represent that this protocol is indeed more effective than many of the others in terms of the product yield, reaction time and using a green solvent.

## Conclusions

In summary, Fe_3_O_4_@CM, the hybrid magnetic material prepared from natural *Chondrus crispus*, was found to be a highly efficient nano-biocatalyst for the synthesis of pyrimidinone derivatives via Biginelli reaction. This method offers several advantages, such as omitting toxic solvents or catalysts, high yields, short reaction time, no waste production, very simple work-up, using a green magnetically separable and recyclable catalyst from a natural source. The elemental composition of the three types of catalysts was analyzed by EDX, which led to the identification of the following main elements in the catalyst structure: C, O, Fe, S and N. The ultrathin coating surrounding the magnetic cores was also evidenced by TEM images.

## Experimental section

### Instruments and characterization

All chemicals were purchased from Merck, Fluka, and Sigma-Aldrich companies and were used without further purification. Thin layer chromatography (TLC) was performed by using aluminum plates coated with silica gel 60 F-254 plates (Merck) using ethyl acetate and *n*-hexane (1:2) as eluents. The spots were detected either under UV light or by placing in an iodine chamber. Melting points were determined in open capillaries using an Electrothermal 9100 instrument. ^1^H NMR (300 MHz) and ^13^C NMR (75 MHz) spectra were recorded on a Bruker Avance DPX-300 instrument. The spectra were measured in DMSO-d*6* relative to TMS as internal standard. FT-IR spectra was obtained with a shimadzu 8400S with spectroscopic grade KBr. Transmission Electron Microscopy characterization of Fe_3_O_4_@CM was performed using a transmission microscope Philips CM-30 with an accelerating voltage of 150 and 250 kV. Scanning electron microscopy (SEM) was recorded on a VEG//TESCAN with gold coating, and energy dispersive X-ray spectroscopy (EDX) was recorded on a VEG//TESCAN-XMU. The TOPSONIC ultrasonic homogenizer was used to perform reactions under ultrasonic irradiation.

### The synthesis of Fe_3_O_4_@CM

Irish moss (0.2 g) was dissolved in distilled water (10 ml), then FeCl_3_.6H_2_O (0.5 g, 1.8 mmol) and FeCl_2_.4H_2_O (0.2 g, 1 mmol) was added to the solution. The mixture was stirred at 80 °C, until obtaining a clear solution and then aqueous ammonia (25%) was added to this solution until the medium reached pH 12. The solution was maintained at 80 °C under vigorous stirring for 30 min. The precipitate was collected with an external magnet, and washed with water and methanol for several times, then dried under vacuum.

### General procedure for the synthesis of pyrimidinone derivatives

In a 50 ml round-bottom flask, a mixture of an aromatic aldehyde (1 mmol), urea or thiourea (1 mmol), a β-ketoester (1 mmol) and Fe_3_O_4_@CM (10 mg) was refluxed in H_2_O (3 ml). After completion of the reaction, as indicated by TLC, the Fe_3_O_4_@CM was separated with an external magnet and then the product was purified by recrystallization in hot ethanol.

### Spectra data for the synthesis compounds (4a, 4f, 4i and 4m)

#### Ethyl 4-(4-chlorophenyl)-1,2,3,4-tetrahydro-6-methyl-2-oxopyrimidine-5-carboxylate (4a)

IR (KBr): ν (cm^−1^) 3241, 3114, 2968, 1713, 1645, 1469; mp (^o^C):208–210; ^1^H NMR (300 MHz-DMSO-d*6*): δ (ppm): 1.19 (t, 3H), 2.36 (s, 3H, CH3), 4.10 (q, 2H, CH_2_), 5.40 (d, 1H, CH), 5.72 (s, 1H, NH), 7.26–7.32 (m, 4H, Ar–H), 7.76 (brs, 1H), 9.23 (brs, 1H); ^13^C NMR (75 MHz, DMSO-d*6*): δ (ppm): 14.1, 17.8, 53.2, 60.1, 101.1, 128.0, 128.9, 133.7, 142.1, 146.3, 152.9, 165.4.

#### Methyl 1,2,3,4-tetrahydro-6-methyl-2-oxo-4-phenylpyrimidine-5-carboxylate (4f)

IR (KBr): v (cm^−1^) 3332, 3224, 3107, 2947, 1706, 1668; mp (^o^C): 233–235; ^1^H NMR (300 MHz, DMSO-*d6*) δ ppm = 2.25 (s, 3H), 3.53 (s, 3H), 5.14 (s, 1H), 7.33–7.23 (m, 5H, Ar–H), 7.74 (brs, 1H, NH), 9.21 (brs, 1H, NH); ^13^ CNMR (75 MHz, DMSO-*d*6, δ ppm): 165.8, 152.1, 148.6, 144.6, 128.4, 127.2, 126.1, 99.0, 53.7, 50.7, 17.8.

#### Methyl 4-(4-chlorophenyl)-1,2,3,4-tetrahydro-6-methyl-2-thioxopyrimidine-5-carboxylate (4i)

IR (KBr): ν (cm^−1^): 3315.41 and 3282.62 (N–H str), 1616.24 (C=O str), 1490.87 (C=S), 1413.12 (C–N), 1085.85 (C–O), 717.47 (C–Cl), ^1^HNMR (300 MHz-DMSO-d*6*), δ (ppm): 2.42 (s, 3H), 3.51 (s, 3H), 5.32 (s, 1H), 7.22 (d, 2H, J = 8 Hz, Ar–H), 7.41 (d, 2H, J = 8 Hz, Ar–H), 9.18 (s, 1H), 9.75 (S, 1H); ^13^CNMR (75 MHz, DMSO-*d*6), δ (ppm): 21.1, 50.4, 60.3, 108.4, 125.2, 128.4, 134.4, 143.1, 156.6, 170.3, 175.5.

#### Ethyl 1,2,3,4-tetrahydro-6-methyl-4-(3-nitrophenyl)-2-thioxopyrimidine-5-carboxylate (4m)

IR (KBr, cm^−1^): 3360.98 and 3276.83 (N–H str), 1640 (C=O str), 1471.59 (C–S), 1413.72 (C–N and N=O, overlap and str), 1083.92 (C–O), ^1^HNMR, (300 MHz-DMSO-d*6*), δ (ppm): 1.40 (t, J = 7.2 Hz, 3H), 2.28 (s, 3H), 4.76 (q, J = 7.2 Hz, 2H), 5.35 (s, 1H), 7.61–8.22 (m, 4H), 9.12 (s, 1H), 9.84 (s, 1H); ^13^CNMR, (75 MHz, DMSO-d*6*) δ (ppm): 16.2, 19.23, 57.4, 61.3, 103.4, 120.5, 122.3, 127.7, 133.2, 142.5, 148.6, 161, 168.3, 173.3.

## Additional file

**Additional file 1: Figure S1.** FT-IR Spectra of Fe_3_O_4_@CM. **Figure S2.** XRD analysis of Fe_3_O_4_@CM. **Figure S3.** SEM micrograph of Fe_3_O_4_@CM. **Figure S4.** TEM Micrograph of Fe_3_O_4_@CM. **Figure S5.** VSM analysis of Fe_3_O_4_ and Fe_3_O_4_@CM. **Figure S6.** EDX analysis of Fe_3_O_4_@CM. **Figure S7.** TGA-DTA analysis of Fe_3_O_4_@CM.
