# Supplementary material for: Magnetic core–shell Carrageenan moss/Fe3O4: a polysaccharide-based metallic nanoparticles for synthesis of pyrimidinone derivatives via Biginelli reaction
Source: Chem Cent J. 2018 Oct 27;12:108. doi: 10.1186/s13065-018-0477-3 (PMC6768032; doi:10.1186/s13065-018-0477-3)
Supplement: Supplementary file 1 — Additional file 1: Figure S1. FT-IR Spectra of Fe3O4@CM. Figure S2. XRD analysis of Fe3O4@CM. Figure S3. SEM micrograph of Fe3O4@CM. Figure S4. TEM Micrograph of Fe3O4@CM. Figure S5. VSM analysis of Fe3O4 and Fe3O4@CM. Figure S6. EDX analysis of Fe3O4@CM. Figure S7. TGA-DTA analysis of Fe3O4@CM. [file 13065_2018_477_MOESM1_ESM.doc]

**Additional Information:**

# Magnetic Core-shell Carrageenan Moss/Fe_3_O_4_: A Polysaccharide-Based Metallic Nanoparticles for Synthesis of Pyrimidinone Derivatives via Biginelli Reaction

Hossein Mohammad Zaheri^a^, Shahrzad Javanshir^a^*, Behnaz Hemmati^a^, Zahra Dolatkhah^a^, Maryam Fardpour^a^

*^a^Heterocyclic chemistry Research Laboratory, Department of Chemistry, Iran University of Science and Technology, Tehran 16846-13114, Iran.*

| **Contents** | **Page** |
| --- | --- |
| Figure S1. FT-IR Spectra of Fe_3_O_4_@CM | 2 |
| Figure S2. XRD analysis of Fe_3_O_4_@CM | 3 |
| Figure S3. SEM micrograph of Fe_3_O_4_@CM | 3 |
| Figure S4. TEM Micrograph of Fe_3_O_4_@CM | 4 |
| Figure S5. VSM analysis of Fe_3_O_4_ and Fe_3_O_4_@CM | 4 |
| Figure S6. EDX analysis of Fe_3_O_4_@CM | 5 |
| Figure S7. TGA-DTA analysis of Fe_3_O_4_@CM | 5 |


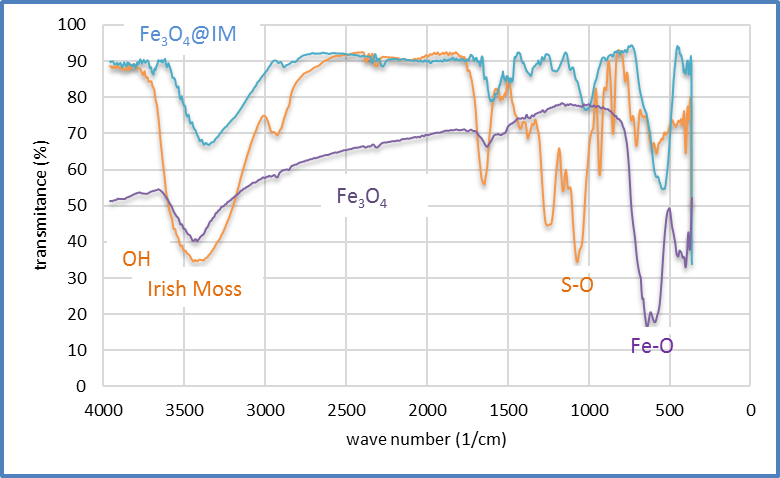


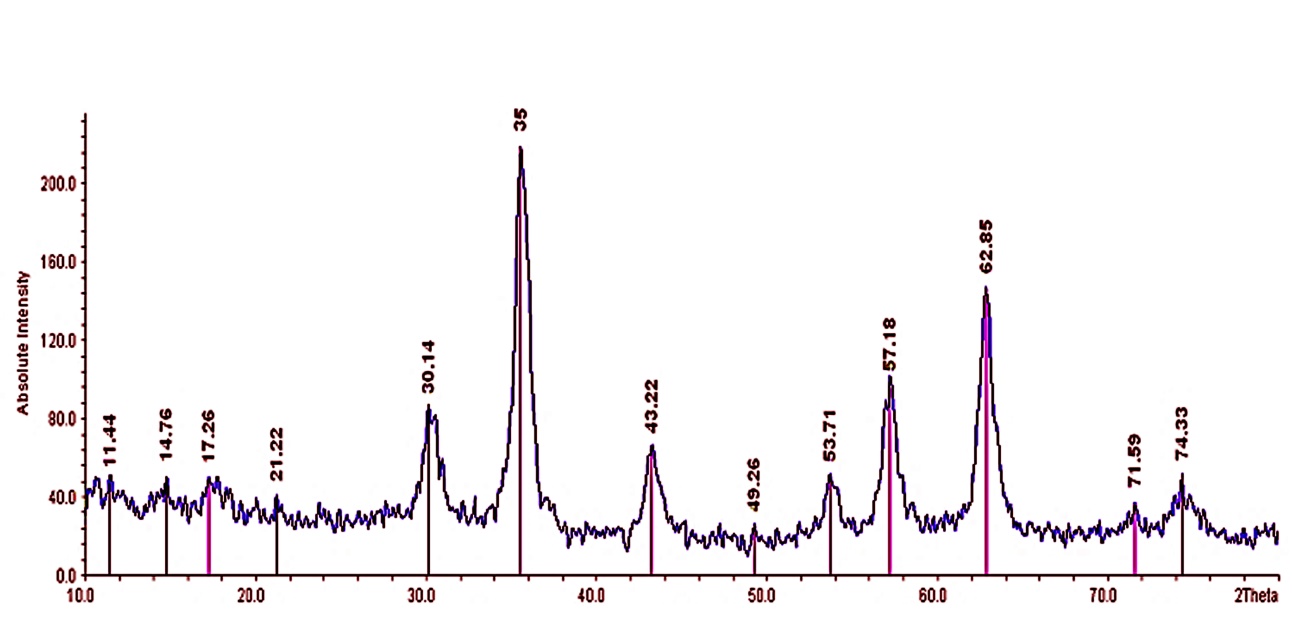
Figure S1. The FT-IR spectra of Carrageenan Moss, Fe_3_O_4_ and Fe_3_O_4_@CM

Figure S2. The XRD analysis of Fe_3_O_4_@CM


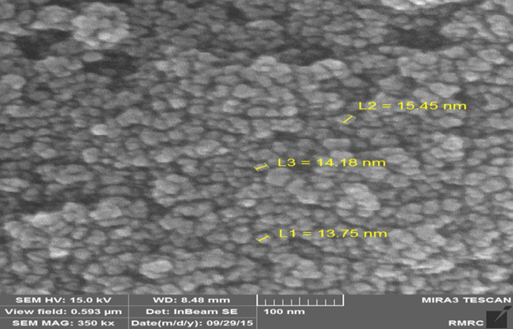


Figure S3. SEM Micrograph of Fe_3_O_4_@CM

**
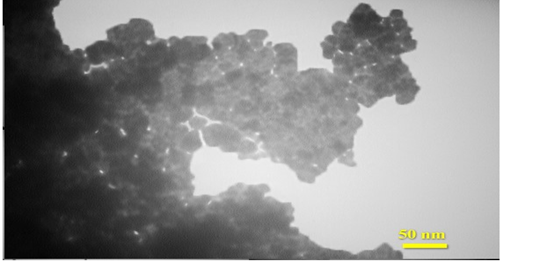
**

Figure S4. TEM Micrograph of Fe_3_O_4_@CM

**
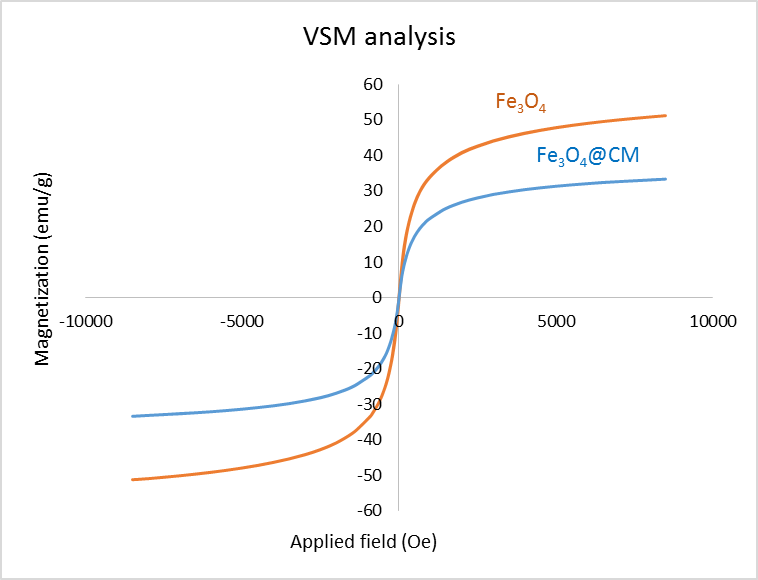
**

Figure S5. VSM analysis of Fe_3_O_4_ and Fe_3_O_4_@CM


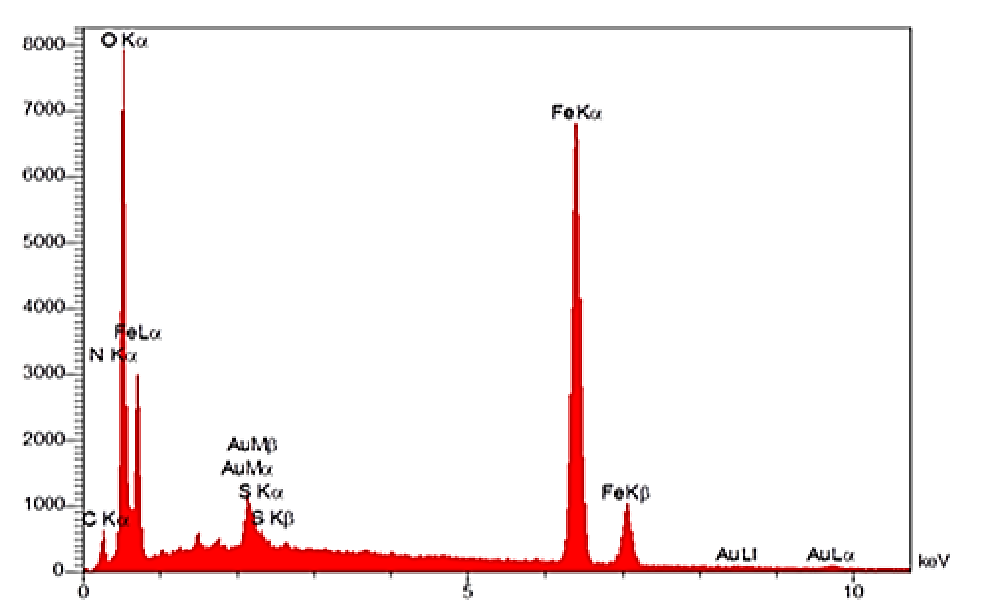


Figure S6. EDX analysis of Fe_3_O_4_@CM


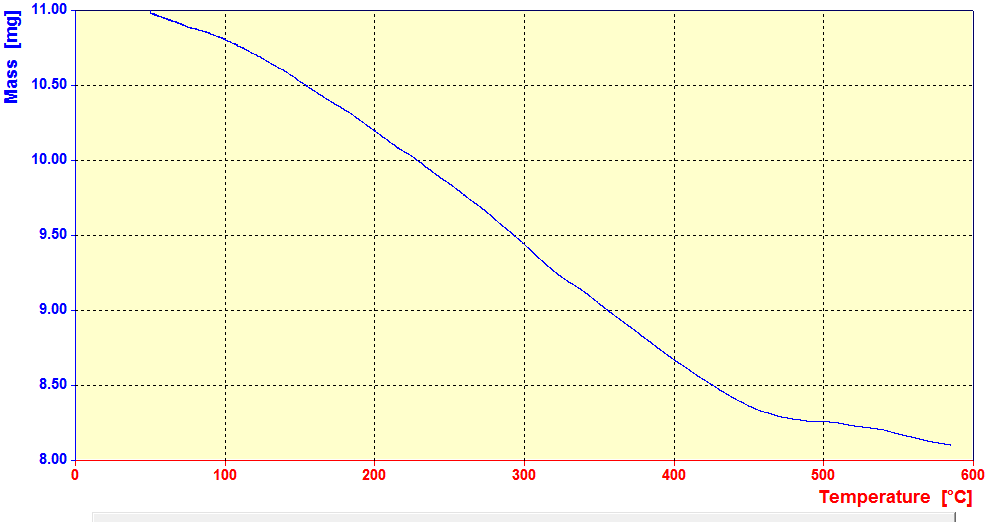


Figure S7. TGA-DTA analysis of Fe_3_O_4_@CM
